# Association of Maternal Iodine Status With Child IQ: A Meta-Analysis of Individual Participant Data

**DOI:** 10.1210/jc.2018-02559

**Published:** 2019-03-28

**Authors:** Deborah Levie, Tim I M Korevaar, Sarah C Bath, Mario Murcia, Mariana Dineva, Sabrina Llop, Mercedes Espada, Antonius E van Herwaarden, Yolanda B de Rijke, Jesús M Ibarluzea, Jordi Sunyer, Henning Tiemeier, Margaret P Rayman, Mònica Guxens, Robin P Peeters

**Affiliations:** 1 The Generation R Study Group, Erasmus University Medical Centre, CA Rotterdam, Netherlands; 2 Department of Internal Medicine, Academic Center For Thyroid Diseases, Erasmus University Medical Centre, CA Rotterdam, Netherlands; 3 Department of Child and Adolescent Psychiatry/Psychology, Erasmus University Medical Centre–Sophia Children’s Hospital, CB Rotterdam, Netherlands; 4 ISGlobal, Barcelona, Spain; 5 Pompeu Fabra University, Barcelona, Spain; 6 Spanish Consortium for Research on Epidemiology and Public Health, Instituto de Salud Carlos III, Madrid, Spain; 7 Department of Nutritional Sciences, Faculty of Health and Medical Sciences, University of Surrey, Guildford, United Kingdom; 8 Epidemiology and Environmental Health Joint Research Unit, FISABIO-Universitat Jaume I-Universitat de València, Valencia, Spain; 9 Clinical Chemistry Unit, Public Health Laboratory of Bilbao, Basque Government, Parque Tecnológico de Bizkaia, Derio, Spain; 10 Department of Laboratory Medicine, Radboud University Nijmegen Medical Centre, GA Nijmegen, Netherlands; 11 Department of Clinical Chemistry, Erasmus University Medical Centre, CN Rotterdam, Netherlands; 12 Departamento de Sanidad Gobierno Vasco, Subdirección de Salud Pública de Guipúzcoa, Donostia – San Sebastián, Spain; 13 BIODONOSTIA Health Research Institute, Donostia – San Sebastián, Spain; 14 Faculty of Psychology, University of the Basque Country UPV/EHU, Donostia – San Sebastián, Spain; 15 Hospital del Mar Research Institute, Barcelona, Spain; 16 Department of Social and Behavioral Science, Harvard TH Chan School of Public Health, Boston, Massachusetts

## Abstract

**Context:**

Although the consequences of severe iodine deficiency are beyond doubt, the effects of mild to moderate iodine deficiency in pregnancy on child neurodevelopment are less well established.

**Objective:**

To study the association between maternal iodine status during pregnancy and child IQ and identify vulnerable time windows of exposure to suboptimal iodine availability.

**Design:**

Meta-analysis of individual participant data from three prospective population-based birth cohorts: Generation R (Netherlands), INMA (Spain), and ALSPAC (United Kingdom); pregnant women were enrolled between 2002 and 2006, 2003 and 2008, and 1990 and 1992, respectively.

**Setting:**

General community.

**Participants:**

6180 mother-child pairs with measures of urinary iodine and creatinine concentrations in pregnancy and child IQ. Exclusion criteria were multiple pregnancies, fertility treatment, medication affecting the thyroid, and preexisting thyroid disease.

**Main Outcome Measure:**

Child nonverbal and verbal IQ assessed at 1.5 to 8 years of age.

**Results:**

There was a positive curvilinear association of urinary iodine/creatinine ratio (UI/Creat) with mean verbal IQ only. UI/Creat <150 µg/g was not associated with lower nonverbal IQ (−0.6 point; 95% CI: −1.7 to 0.4 points; *P* = 0.246) or lower verbal IQ (−0.6 point; 95% CI: −1.3 to 0.1 points; *P* = 0.082). Stratified analyses showed that the association of UI/Creat with verbal IQ was only present up to 14 weeks of gestation.

**Conclusions:**

Fetal brain development is vulnerable to mild to moderate iodine deficiency, particularly in the first trimester. Our results show that potential randomized controlled trials investigating the effect of iodine supplementation in women with mild to moderate iodine deficiency on child neurodevelopment should begin supplementation not later than the first trimester.

## Teaser

This meta-analysis of individual participant data showed that a lower urinary iodine/creatinine ratio during pregnancy was associated with lower verbal IQ up to the 14th week of gestation.

Iodine is an essential trace element required for the production of thyroid hormones; optimal thyroid hormone availability is important for normal fetal brain development (1, 2). During pregnancy, there is a higher demand for maternal iodine intake (3, 4). This is due to (i) the increased maternal thyroid hormone synthesis required to ensure adequate thyroid hormone availability to the fetus, (ii) greater urinary iodine loss due to an increased glomerular filtration rate, and (iii) placental transfer of iodine to the fetus to facilitate fetal thyroid hormone production. Although severe iodine deficiency is no longer common in Europe, mild to moderate iodine deficiency is still common, especially in pregnant women (5). Severe iodine deficiency in pregnancy results in a higher risk of goiter, hypothyroidism, and mental retardation in the offspring (6). However, the consequences of mild to moderate iodine deficiency in pregnancy on child neurodevelopment are less well established (4).

Mild to moderate iodine deficiency or low iodine intake during pregnancy has been associated with adverse child neurodevelopmental outcomes in some (7–13) but not all studies (14–16). Differences in results between studies may be related to methodological differences (*e.g.,* measurement of iodine status, selected reference group, and available data on confounders), the age at assessment of the neurodevelopmental outcome of interest, the timing of the iodine measurements, and the relative severity of iodine deficiency in the population. Although the main focus in the literature has been on the effects of iodine deficiency, some studies have suggested adverse effects of supplemental intake or excess iodine on either maternal thyroid function (17, 18), fetal thyroid function (19, 20), or child neurodevelopment (10, 15, 16).

International health authorities have similar recommendations to ensure optimal iodine status in pregnancy (21–23). It is universally recognized that any necessary iodine supplementation should be commenced before or as early as possible in pregnancy to achieve adequate iodine intake, owing to the susceptibility of the fetal brain to iodine deficiency (23). However, whether the effect of iodine on child cognition varies during different stages of pregnancy is unknown. We therefore assessed the association between maternal iodine status in pregnancy and child IQ across three cohorts of differing iodine status and investigated potential effect modification by gestational age.

## Material and Methods

### Study design and populations

This study was embedded in three cohort studies: Generation R (Netherlands), the INfancia y Medio Ambiente Project (INMA; Spain, three regions), and the Avon Longitudinal Study of Parents and Children (ALSPAC; United Kingdom). The study designs have been described elsewhere (24–27); the ALSPAC study website contains details of all the data that are available through a fully searchable data dictionary and variable search tool (28). For the current study, mother-child pairs were included if a measure of urinary iodine and creatinine concentration during pregnancy and child IQ scores were available. Exclusion criteria were multiple pregnancies, fertility treatment, medication affecting the thyroid, and preexisting thyroid disease. Ethical approval was obtained from the Medical Ethical Committee of the Erasmus Medical Center (Generation R); the Ethical Committee of the Municipal Institute of Medical Investigation and the ethical committees of the hospitals involved in the study (INMA); and the ALSPAC Ethics and Law Committee and local research ethics committees; approval was given by participants and/or parents or guardians of the children by a signed informed consent form.

### Maternal iodine status

Urinary iodine concentration (UIC) and creatinine concentration were measured in spot urine samples stored at −20°C after collection. As part of this study, additional urine samples were analyzed for iodine and creatinine concentrations, and existing measurements from each cohort (7, 19, 29) were also included. The additional measurements were performed in the same laboratories where the existing measurements were performed. The laboratories were registered with EQUIP and used certified reference materials (Seronorm Urine levels one and two; Nycomed, Norway) for the verification of results. In Generation R, UIC was measured by the Sandell-Kolthoff method. In the INMA, UIC was measured using paired-ion reversed-phase, high-performance liquid chromatography with electrochemical detection at a silver working electrode (Waters Chromatography, Milford, MA). In the ALSPAC, UIC was measured on a dynamic reaction cell inductively coupled plasma mass spectrometer. Urinary creatinine concentration was determined by the Jaffe rate method in all cohorts. More information on the measurement methods and the variability between assays can be found in an online repository (30).

In a subset of women, repeated measures of urinary iodine and creatinine were available; we used the earliest available sample as an indicator of iodine status. The urinary iodine/creatinine ratio (UI/Creat) was used as a measure of iodine status. Because of possible contamination of UIC by the use of iodine-containing test strips in ALSPAC (31), UIC >500 µg/L and/or UI/Creat >700 µg/g was excluded from the analyses in this cohort (N = 363). These cutoffs were based on previous work in ALSPAC and from other studies of pregnant women in the United Kingdom (7, 32, 33). We grouped women’s results by UI/Creat as follows: (i) <150 µg/g, (ii) 150 to <500 µg/g, and (iii) ≥500 µg/g; according to World Health Organization classification, these groups broadly relate to iodine deficiency, sufficiency, and excess, respectively.

### Maternal thyroid function

TSH and free thyroxine (FT4) were measured according to different methodologies between cohorts, which are described in detail elsewhere (34–36). For the analysis, FT4 and TSH concentrations were logarithmically transformed, and cohort-specific SD scores were calculated with a mean of 0 and an SD of 1 based on the data of thyroid peroxidase antibody (TPOAb)-negative women (TPOAb measurements were available in Generation R and ALSPAC). TPOAb titers ≥60 IU/mL and ≥6 IU/mL were considered positive in Generation R and ALSPAC, respectively. These cutoffs were determined by the assay manufacturers.

### Nonverbal and verbal IQ scores

In Generation R, nonverbal IQ was assessed at a median age of 5.9 years using a subset of the Snijders Oomen Nonverbal Intelligence Test (2.5-7-Revised) (37), and verbal IQ was estimated by the short form of the McArthur Communicative Development Inventory (38) at a median age of 1.5 years. In the INMA, nonverbal and verbal IQ scores were assessed at a median age of 4.6 years using the McCarthy Scales of Children’s Abilities (39). In the ALSPAC, nonverbal and verbal IQ scores were assessed at a median age of 8.6 years using the Wechsler Intelligence Scale for Children, third UK edition (40). Except for verbal IQ ascertainment in Generation R, which involved a parental questionnaire, all other measurements were performed by psychologists or trained staff. To homogenize the different scores, raw cohort-specific scores were standardized to a mean of 100 and an SD of 15. Children with IQ scores <50 or >150 (n = 3) were considered outliers and were excluded from the analyses. Suboptimal IQ was defined as an IQ score <85.

### Potential confounding variables

Information on maternal age, educational level (low, middle, high), ethnicity/country of birth (cohort-specific categories), parity (zero, one, two or more), prepregnancy body mass index, and smoking during pregnancy (never smoked, smoked in the beginning or until pregnancy confirmed, continued smoking) was collected by questionnaires administered during pregnancy. Gestational age at urine sampling was defined using ultrasonography or last menstrual period. Child sex and age at time of the IQ assessment was obtained during the study visits.

### Statistical analyses

UI/Creat was not normally distributed and was therefore transformed using the natural logarithm; back-transformed values are shown in plots for better interpretation. We studied the associations of UI/Creat with child nonverbal and verbal IQ scores by using one-step and two-step approaches. In the one-step approach, data from the cohorts were pooled, and we performed standard multivariable linear regression models with and without a quadratic term to investigate the possible nonlinear nature of the associations. Nonlinearity was also investigated by using ordinary least squares linear regression models with restricted cubic splines with three knots. With ANOVA, we tested the null hypothesis that child mean IQ was similar across the full range of the natural logarithm of UI/Creat. The decision to use linear regression models instead of multilevel models for the one-step analyses was made because we found no difference between multilevel models with random intercepts and/or slope per cohort vs standard linear regression correcting for cohort (*e.g.,* cohort-specific variable ethnicity/country of birth) when assessed using the Akaike information criterion and log-likelihood tests. In the two-step approach we first studied the associations of UI/Creat <150 µg/g and UI/Creat ≥500 µg/g with child IQ by using linear regression models in each cohort separately. In these analyses, the reference group consisted of women with UI/Creat of 150 to 500 µg/g. We then combined the cohort-specific effect estimates using random-effects meta-analyses.

Potential effect modification according to gestational age was analyzed by adding a product interaction term between UI/Creat and gestational age to the one-step approach models. Because of the known constraints of statistical power for interaction analyses, a *P* value <0.15 for interaction terms was used to screen for potential relevant modification (41). We further quantified potential relevant differences by performing stratified analyses by tertiles of gestational age (≤12 weeks, >12 to ≤14 weeks, and >14 weeks). We also studied associations with suboptimal IQ (score <85) by combining cohort-specific estimates from logistic regression models into random-effects meta-analyses.

Sensitivity analyses were designed to study (i) the associations of UI/Creat with verbal IQ score in mother-child pairs from the INMA and ALSPAC only, as verbal IQ was assessed at a preschool age in Generation R; (ii) the association between UI/Creat and maternal TSH and FT4 SD scores within the ±4 SD range around the mean, as TSH and FT4 values outside this range were considered outliers (n = 19 for TSH; n = 5 for FT4); and (iii) whether the association between UI/Creat and IQ score could potentially be explained by maternal thyroid function by adjusting for FT4 and TSH in the models.

Heterogeneity between cohorts was assessed using the Cochran Q test and the I^2^ statistic (42). All models were adjusted for potential confounding variables. However, because of collinearity between maternal ethnicity/country of birth, child age at IQ ascertainment, and cohort, we adjusted for maternal ethnicity/country of birth only in the one-step approach models.

We applied inverse probability weighting to take into account the potential differential loss to follow-up (30) [*i.e.,* to account for selection bias that potentially arises when only the population with available data on iodine status and child IQ is included compared with a full initial cohort recruited at pregnancy (43)]. Briefly, we used information available for all participants at recruitment to predict the probability of participation in the study and used the inverse of these probabilities as weights in the analyses so that results would be representative of the initial populations of the cohorts. In addition, missing values in potential confounding variables were imputed using chained equations (44). A total of 25 data sets were generated. A *P* value <0.05 was defined as statistically significant. Statistical analyses were performed with STATA (version 14.0; StataCorp, College Station, TX) and R statistical software (version 3.3.2, package rms).

## Results

**Figure 1.**
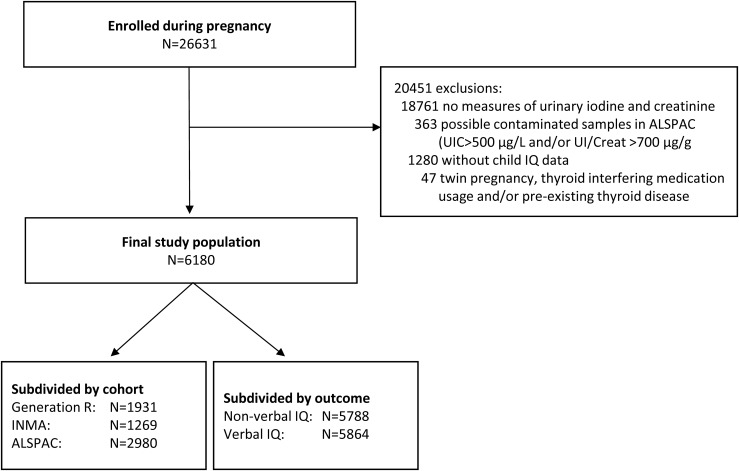
Flowchart of selection of the study population.

The final study population consisted of 6180 mother-child pairs (Fig. 1). The median UIC (UI/Creat) was 159 µg/L (214 µg/g) in Generation R (adequate intake), 128 µg/L (152 µg/g) in the INMA (mild deficiency), and 96 µg/L (124 µg/g) in the ALSPAC (moderate deficiency) (Table 1). Iodine status was determined at a median [interquartile range (IQR)] gestational age of 13.1 (12.1, 14.8) weeks, 13.0 (12.4, 14.1) weeks, and 12.0 (8.0, 16.0) weeks in Generation R, the INMA, and the ALSPAC, respectively.

**Table 1.** Population Characteristics

	Generation R (n = 1931)		INMA (n = 1269)		ALSPAC (n = 2980)	
	n	Values	n	Values	n	Values
Offspring neurodevelopment, no. (%)						
Suboptimal nonverbal IQ^*a*^	1540	175 (11.4)	1269	216 (17.0)	2979	479 (16.1)
Suboptimal verbal IQ^*a*^	1618	279 (17.2)	1269	211 (16.6)	2977	480 (16.1)
Female sex, no. (%)	1931	963 (49.9)	1268	632 (49.8)	2980	1514 (50.8)
Iodine status	1931		1269		2980	
UI/Creat, µg/g, median (IQR)		214 (143–308)		152 (96–258)		124 (82–199)
UI/Creat <150 µg/g, no. (%)		531 (27.5)		623 (49.1)		1831 (61.4)
UI/Creat >500 µg/g, no. (%)		97 (5.0)		52 (4.1)		81 (2.7)
UIC, µg/L, median (IQR)		159 (90–275)		128 (75–213)		96 (57–153)
Gestational age at urine sampling, wk	1931		1267		2980	
Median (IQR)		13.1 (12.1–14.8)		13.0 (12.4–14.1)		12.0 (8.0–16.0)
Range (min–max)		6.1–30.5		8.6–39.4		1.0–42.0
>20th week of gestation, no. (%)		66 (3.4)		130 (10.2)		211 (7.1)
Maternal thyroid function						
TSH, mIU/L, median (IQR)	1719	1.29 (0.79–1.95)	1227	1.25 (0.85–1.80)	1102	0.97 (0.64–1.38)
FT4, pmol/L, median (IQR)	1728	14.6 (12.9–16.5)	1229	10.6 (9.7–11.6)	1108	16.2 (14.9–17.7)
TPOAb positivity, no. (%)	1737	98 (5.6)	NA	NA	1111	146 (13.1)
Gestational age, wk, mean (SD)	1733	13.3 (1.9)	1228	13.2 (1.4)	1118	10.3 (2.7)
Educational level, no. (%)	1835		1265		2888	
Low		154 (8.4)		270 (21.3)		573 (19.8)
Middle		760 (41.4)		525 (41.5)		1810 (62.7)
High		921 (50.2)		470 (37.2)		505 (17.5)
Maternal ethnicity/country of birth, no. (%)	1803		1266		2877	
Spanish		NA		1184 (93.5)		NA
Latin-American		NA		56 (4.4)		NA
European/other		NA		26 (2.1)		NA
Dutch		1012 (53.2)		NA		NA
Indonesian		69 (3.6)		NA		NA
Cape Verdean		58 (3.1)		NA		NA
Moroccan		115 (6.0)		NA		NA
Surinamese		154 (8.1)		NA		NA
Turkish		170 (9.0)		NA		NA
Other, non-Western		150 (7.9)		NA		NA
Other, Western		174 (9.1)		NA		NA
White		NA		NA		2841 (98.7)
Nonwhite		NA		NA		36 (1.3)
Maternal age, y, mean (SD)	1931	30.5 (4.8)	1257	31.6 (3.9)	2980	28.6 (4.5)
Parity, no. (%)	1931		1267		2877	
0		1121 (58.1)		727 (57.4)		1346 (46.8)
1		564 (29.2)		458 (36.1)		992 (34.5)
≥2		246 (12.7)		82 (6.5)		539 (18.7)
Smoking during pregnancy, no. (%)	1744		1254		2926	
Never		1319 (75.6)		870 (69.4)		2434 (83.2)
In the beginning of pregnancy		168 (9.6)		168 (13.4)		125 (4.3)
Continued		257 (14.7)		216 (17.2)		367 (12.5)
Prepregnancy BMI, kg/m^2^, median (IQR)	1694	22.6 (20.8–25.2)	1269	22.5 (20.8–25.0)	2713	22.2 (20.5–24.4)

Values are based on unimputed data.

Abbreviations: BMI, body mass index; IQR, interquartile range; NA, not applicable.

^a^ Suboptimal is defined as an IQ score <85.

### Nonverbal IQ

**Figure 2.**
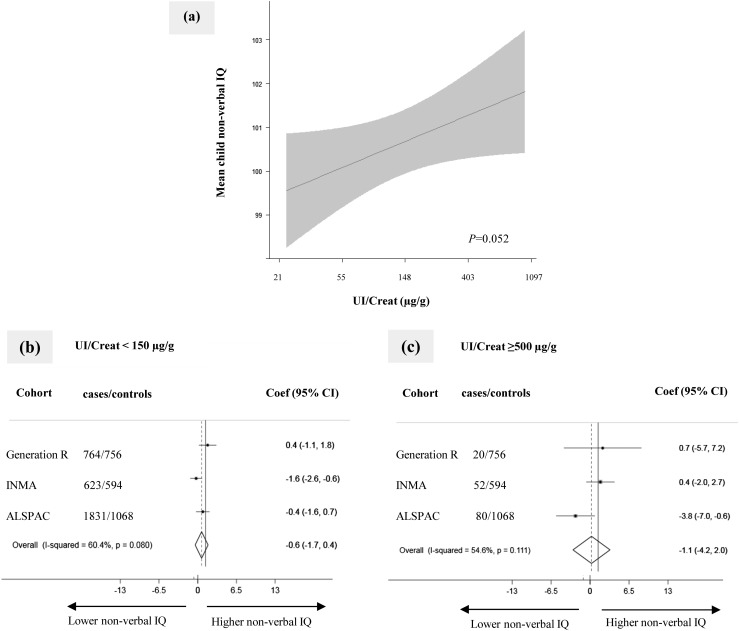
Association of maternal iodine status in pregnancy with child nonverbal IQ score. (a) Continuous association, depicted as the mean child nonverbal IQ score (black line) with 95% CI (gray area) using pooled data. Models were adjusted for gestational age, child sex, maternal ethnicity/country of birth, maternal education, parity, maternal age, prepregnancy body mass index, and smoking during pregnancy. The *P* value was provided by an ANOVA test of the null hypothesis that mean child nonverbal IQ score was similar across the whole range of the natural logarithm of UI/Creat. (b and c) Forest plots of (b) UI/Creat <150 µg/g (“deficiency”) and (c) UI/Creat ≥500 µg/g (“excess”) compared with the reference group of UI/Creat ≥150 to <500 µg/g (“sufficient”), depicted as effect estimate (dot) with 95% CI per cohort and overall as estimated by random-effects meta-analysis (diamond). Coef, coefficient.

Using pooled data in the one-step approach, we observed a positive linear association between the UI/Creat and mean nonverbal IQ score [Fig. 2(a) and (30)], although this association was not statistically significant. Using the two-step approach in which we combined cohort-specific effect estimates using random-effects meta-analysis, neither UI/Creat <150 µg/g nor UI/Creat ≥500 µg/g was associated with nonverbal IQ score (−0.6 point; 95%CI: −1.7 to 0.4 points; *P* = 0.246 and −1.1 points, 95%CI: −4.2 to 2.0 points; *P* = 0.478) [Fig. 2(b) and 2(c)]. UI/Creat was not associated with suboptimal nonverbal IQ (30).

### Verbal IQ

**Figure 3.**
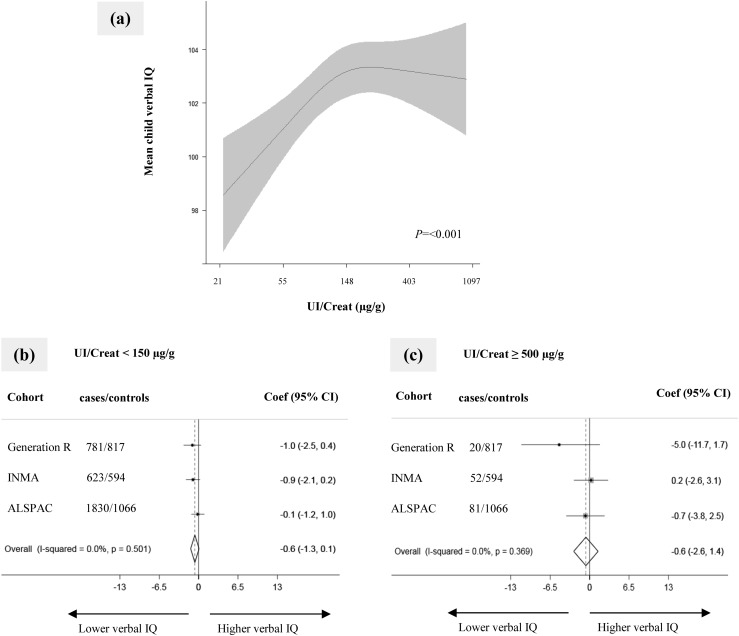
Association of maternal iodine status during pregnancy with child verbal IQ score. (a) Continuous association, depicted as the mean child verbal IQ (black line) with 95% CI (gray area) using pooled data. Models were adjusted for gestational age, child sex, maternal ethnicity/country of birth, maternal education, parity, maternal age, prepregnancy body mass index, and smoking during pregnancy. The *P* value was provided by an ANOVA test of the null hypothesis that mean child verbal IQ score was similar across the whole range of the natural logarithm of UI/Creat. (b and c) Forest plots of (b) UI/Creat <150 µg/g (“deficiency”) and (c) UI/Creat ≥500 µg/g (“excess”) compared with the reference group of UI/Creat ≥150 to <500 µg/g (“sufficient”), depicted as effect estimate (dot) with 95% CI per cohort and overall as estimated by random-effects meta-analysis (diamond). Coef, coefficient.

Using the one-step approach, we observed a positive curvilinear association between UI/Creat and verbal IQ score [Fig. 3(a) and (30)]. There was a positive linear association when measures in preschool children from Generation R were excluded. Using the two-step approach, neither UI/Creat <150 µg/g nor UI/Creat ≥500 µg/g was associated with verbal IQ score (−0.6 point, 95% CI: −1.3 to 0.1 points; *P* = 0.082 and −0.6 point, 95% CI: −2.6 to 1.4 points; *P* = 0.552, respectively) [Fig 3(b) and 3(c) or suboptimal verbal IQ score (30)].

### Effect modification according to gestational age

**Figure 4.**
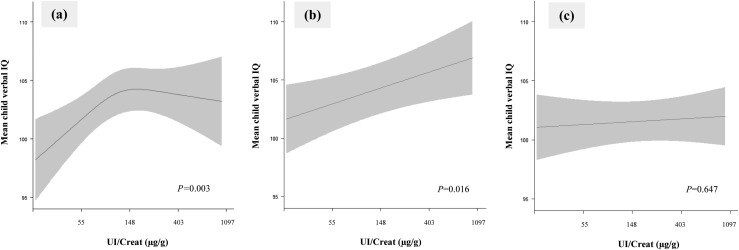
Association of maternal iodine status during pregnancy with child verbal IQ score stratified by tertiles of gestational age. Continuous association, depicted as the mean child verbal IQ (black line) with 95% CI (gray area) was restricted to (a) the first 12 weeks of gestation (lowest tertile, median UI/Creat 116 µg/g; n = 2209); (b) from weeks 12 to 14 of gestation (middle tertile, median UI/Creat 147 µg/g; n = 1776); and (c) later than week 14 of gestation (highest tertile, median UI/Creat 157 µg/g; n = 1879). Models are adjusted for gestational age, child sex, maternal ethnicity/country of birth, maternal education, parity, maternal age, prepregnancy body mass index, and smoking during pregnancy. The *P* value was provided by an ANOVA test of the null hypothesis that mean child verbal IQ was similar across the whole range of the natural logarithm of UI/Creat.

The continuous association of UI/Creat with nonverbal IQ score did not differ according to gestational age at measurement *(P for interaction term =* 0.306*).* By contrast, we identified possible effect modification by gestational age in the association with verbal IQ (*P for interaction term* = 0.078). Stratification by tertile of gestational age showed a positive curvilinear association of UI/Creat with mean child verbal IQ score, with an overall effect of ∼5 IQ points during the first 12 weeks of pregnancy [Fig. 4(a) and (30)]. Furthermore, there was a positive linear association between UI/Creat and mean child verbal IQ score during the 12th to 14th weeks of pregnancy, with an overall effect of ∼3 IQ points [Fig. 4(b)]. This association was no longer present after the 14th week of pregnancy [Fig. 4(c)].

### Iodine status and thyroid function

UI/Creat was not associated with TSH (0.007, 95 CI%: −0.044 to 0.058; *P* = 0.789) or with FT4 (−0.044, 95 CI%: −0.092 to 0.005; *P* = 0.079). The association did not change between UI/Creat and child nonverbal or verbal IQ score after adjustment for TSH and/or FT4; there was also no sign of effect modification by TSH or FT4 (data not shown). There was no association of UI/Creat with TSH and FT4 in TPOAb-negative women only.

## Discussion

This meta-analysis of individual participant data showed that a lower UI/Creat during pregnancy was associated with lower verbal IQ score. The association of UI/Creat with verbal IQ score was only seen up to the start of the second trimester (up to the 14th week of gestation). In contrast, we observed no associations between IQ score and UI/Creat <150 µg/g or >500 µg/g.

Only a few of the previous single-center studies (*i.e.,* the Generation R and ALSPAC cohort studies) focused on child nonverbal IQ score (7, 14). They found no association between UI/Creat <150 µg/g and nonverbal IQ score. It was suggested that iodine deficiency in the Generation R cohort may not have been severe enough for an association to be identified (14). After combining these two cohorts of contrasting iodine status with a third mildly deficient population (INMA), there was still no effect of iodine deficiency on nonverbal IQ score.

Our meta-analysis using predefined cutoffs showed that UI/Creat <150 µg/g was not associated with lower verbal IQ score. The estimates we found for the ALSPAC contrasted with the strong negative association of maternal UI/Creat <150 µg/g in the first trimester (defined as ≤13 weeks’ gestation) with child verbal IQ score found in a previously published study from that cohort (fully adjusted: −2.9, 95CI%: −5.0 to −0.8; *P* = 0.006) (7). However, there are a few important differences between the studies. Compared with the previous publication, the ALSPAC data in our study included a larger number of mother-child pairs (2980 vs 958), fewer iodine-deficient women [1831 (61.4%) vs 646 (67.4%)], a study population with a higher UI/Creat [median UI/Creat (IQR): 124 (82 to 199) vs 110 (74 to 170)], and most notably, a higher number of mothers with iodine status measured after the first trimester [1135 (38%) vs 0 (0%); median gestational age (IQR): 12 (8 to 16) weeks vs 10.0 (9 to 12) weeks]. In addition, we adjusted our analysis for a more stringent selection of variables. Comparison between study populations and additional analysis of the association between UI/Creat <150 µg/g and a verbal IQ score in the bottom quartile in the whole cohort and in samples from ≤13 weeks’ gestation are described in an online repository (30).

The importance of iodine status in the preconceptional stage for child IQ has recently been shown (45). In early pregnancy, the fetus is fully dependent on the placental transfer of thyroid hormone to support the crucial processes of brain development (2). There is a need for optimal iodine supply from the initiation of conception, implying that sufficient intrathyroidal iodine stores at the preconception stage may well be critical. Indeed, our results suggest that the fetus is particularly sensitive to suboptimal iodine status in the early stages of pregnancy (*e.g.,* ≤14 weeks of gestation) for optimal development of verbal IQ. Effects on verbal IQ could possibly be explained by the impact of mild iodine deficiency, via thyroid hormone, on the auditory system (13, 46). In our study, we did not find evidence that the association between UI/Creat and verbal IQ was mediated via maternal thyroid function. Possible explanations could be that urinary iodine excretion is a highly volatile and crude measurement of individual iodine status and/or a crude marker of thyroidal iodine availability. Alternatively, it is also possible that the effects are (in part) mediated via fetal thyroid function.

This study confirms that low iodine status is associated with a reduction in verbal IQ scores, putting these children at potential risk for poorer academic achievement (47). Furthermore, our findings may have implications on a national level (*e.g.,* by negatively affecting economic growth) (48). However, there is still inconclusive evidence that supplementation in pregnant women with mild to moderate iodine deficiency is beneficial for child neurodevelopment (11, 15, 16, 49–53). A recent randomized placebo-controlled trial showed no benefit on children’s nonverbal or verbal IQ score with daily supplementation with 200 µg of iodine (as potassium iodide) in women with mild iodine deficiency (52). In addition to the already mentioned limitations of that trial (54), our results provide an explanation for the null finding; the trial randomly assigned women at up to 14 weeks of gestation, whereas we showed that maternal iodine status is particularly important in the first trimester. Although our study needs replication, it suggests that the trial might have missed a critical period of vulnerability in women with iodine deficiency. Our results clearly suggest that additional randomized controlled trials should start with iodine supplementation early in the first trimester or preferably even before pregnancy.

The strengths of our study are as follows: A consistent approach to the analysis and harmonization of potential confounding variables across cohorts optimized comparisons; advanced statistical methods were used to overcome selection bias due to loss to follow-up and missing data; and UI/Creat was used as a marker of iodine status. The latter has been shown to be a more valid measure of iodine excretion when used in groups of the same age and sex (55), though we recognize that a single measure may not be reflective of overall iodine status in an individual. A limitation of the study is that the assessment of IQ was performed with different tools at different ages. Nevertheless, the tools measured the same construct (nonverbal or verbal IQ), and the standardization of IQ scores in each cohort facilitated comparison of results across cohorts. Sensitivity analysis in older children only (*e.g.,* excluding children from Generation R, thus reducing the age range at which verbal IQ was assessed) confirmed the association between UI/Creat and verbal IQ score. Another limitation is that UIC was measured in different laboratories using different assays; it is known that urinary iodine measurements vary between laboratories (56). We used laboratories that were registered with EQUIP, and the use of certified reference materials enabled us to ensure the accuracy of the results.

In conclusion, this study confirms that iodine status in pregnancy is associated with child IQ scores, and results indicate that the development of verbal IQ of the fetus is particularly vulnerable to suboptimal iodine concentration during early pregnancy up until the start of the second trimester. As such, our results suggest that iodine supplementation after the first 14 weeks of pregnancy could be outside the critical period during which iodine availability affects fetal brain development. However, further studies should replicate these data and investigate the effects of iodine supplementation.

## Abbreviations:

ALSPAC: Avon Longitudinal Study of Parents and Children
FT4: free thyroxine
INMA: INfancia y Medio Ambiente Project
IQR: interquartile range
TPOAb: thyroid peroxidase antibody
UIC: urinary iodine concentration
UI/Creat: urinary iodine/creatinine ratio
